# Live sequence charts to model medical information

**DOI:** 10.1186/1742-4682-9-22

**Published:** 2012-06-15

**Authors:** Eric Aslakson, Smadar Szekely, Suzanne D Vernon, Lucinda Bateman, Jan Baumbach, Yaki Setty

**Affiliations:** 1 Poiema, LLC, 375 Chelsea Cir NE, Atlanta, GA, 30307, USA; 2 Department of Computer Science and Applied Mathematics, Weizmann Institute of Science, Rehovot, 76100, Israel; 3 Fatigue Consultation Clinic, 1002 E. South Temple, Suite 408, Salt Lake City, UT, 84102, USA; 4 Max-Planck-Institut für Informatik, Computational Systems Biology, 66123, Saarbrücken, Germany; 5 The CFIDS Association of America, PO Box 220398, Charlotte, NC, 28222-0398, USA; 6 Saarland University, Campus E2.1, Saarbrücken, 66123, Germany

**Keywords:** Medical modeling, Live sequence charts, Computational health informatics

## Abstract

**Background:**

Medical records accumulate data concerning patient health and the natural history of disease progression. However, methods to mine information systematically in a form other than an electronic health record are not yet available. The purpose of this study was to develop an object modeling technique as a first step towards a formal database of medical records.

**Method:**

Live Sequence Charts (LSC) were used to formalize the narrative text obtained during a patient interview. LSCs utilize a visual scenario-based programming language to build object models. LSC extends the classical language of UML message sequence charts (MSC), predominantly through addition of modalities and providing executable semantics. Inter-object scenarios were defined to specify natural history event interactions and different scenarios in the narrative text.

**Result:**

A simulated medical record was specified into LSC formalism by translating the text into an object model that comprised a set of entities and events. The entities described the participating components (i.e., doctor, patient and record) and the events described the interactions between elements. A conceptual model is presented to illustrate the approach. An object model was generated from data extracted from an actual new patient interview, where the individual was eventually diagnosed as suffering from Chronic Fatigue Syndrome (CFS). This yielded a preliminary formal designated vocabulary for CFS development that provided a basis for future formalism of these records.

**Conclusions:**

Translation of medical records into object models created the basis for a formal database of the patient narrative that temporally depicts the events preceding disease, the diagnosis and treatment approach. The LSCs object model of the medical narrative provided an intuitive, visual representation of the natural history of the patient’s disease.

## Background

Medical records are products of doctor-patient discussions that summarize the physical and mental health of the patient, with information being in the form of long textual descriptions that amass the patients’ medical conditions over years. This information can be generalized and abstracted into a questionnaire that follows a diagnostic algorithm, consisting of a series of yes/no questions, which can be described as a decision tree that assess the likelihood that self-reported symptoms fit with a particular diagnosis.

Diagnostic algorithms help to familiarize users with general aspects of the illness. However, they may overlook variations among patients. Therefore, they cannot fully support the study of disease development. To reveal patterns in medical information and records there is a requirement to analyze data from numerous patients systematically. This is essential for diseases such as CFS that are medically unexplained. CFS is a diagnosis of exclusion, based on self-reported information and symptoms described by the patient. There is no objective diagnostic test, no known etiology and the symptoms vary greatly among individual patients [1,2]. A systematic study of complex medically-unexplained illnesses such as CFS could potentially identify common patterns in disease development and provide information concerning possible etiologies. To pursue this direction, there is a requirement to integrate ‘piecemeal’ medical records into a single framework that makes possible a comprehensive view and systematic analysis.

The emerging field of Computational Health Informatics aims to enable a more efficient healthcare analysis to optimize patient care [3-5]. Computational Health Informatics research formalizes medical information into electronic databases, allowing common textural structures to be searched and classified by utilizing text mining techniques and natural language processing toolkits [6-8]. The aim of this is to reveal the likelihood of a patient having a specific condition, and can be used to categorize individuals whose symptoms match those corresponding to a predefined medical diagnosis [9,10]. However, owing to the high level of ‘noise’ in free-text analysis, this direction often fails to generate clean hypotheses, or to identify correlations and dynamic properties in the text.

An additional application of Computational Health Informatics is the development of virtual patients: computer-based simulations that are used to educate and train medical students, and to test medical knowledge and skills. The potential impact of this is vast, with possible applications in medical research and education. However, it is difficult to implement virtual patients owing to significant costs and the requirement for intensive computational resources [11-16]. A complementary application develops continuous improvement of clinical information systems including supportive environments for the daily activity of patients (example ref. [17]).

Herein, a Computational Health Informatics approach is presented that formalizes a narrative obtained from a new patient interview using Live Sequence Charts (LSC) [18]. The LSC is a visual formalism that can be compiled into a machine program to accelerate the analysis of medical information. This methodology provides a platform for translating textual information to a formal specification and enables a platform for a systematic view of multiple medical records to be achieved. LSCs utilize a visual scenario-based programming language to build object models from medical records. The object models provide an intuitive way to read the medical record, and a rapid and formal method to enter and amend data. In contrast with electronic medical records that are static textual descriptions, the LSC object model defines key events that link scenarios on a temporal basis. The scenario-encoding step can be inferred in places where the text is not entirely clear. The translated records are available as a free-format text that is encoded into a machine-readable format to allow further automated analysis to be conducted. Potentially, the object model can reveal relations among diagnosis, disease progression and treatment. The method enables multiple records to be integrated into a single database and provides a platform for future formal analysis of the database. Herein, a conceptual example of a synthetic record is provided to demonstrate the approach, and an object model that specifies scenarios of a record of a real patient is presented. The scenario representation of the medical narrative obtained during the first visit provides an intuitive, visual representation that encapsulates the development of the disease, the diagnosis and treatment.

## Methods and implementation

### A rigorous visual specification for medical object models

LSCs constitute a visual formalism for inter-object scenario-based specification and programming, which extends the classical language of UML message sequence charts (MSC) predominantly through addition of modalities [18]. It allows inter-object scenarios to be defined in order to specify event interactions between entities in individual charts that represent different scenarios [18]. Each LSC consists of a set of entities and a set of interactions that form the scenario. The language offers the ability to specify a set of events that take place and allow a further set of events to take place in the same or other charts. This language is a rigorous formalism that can be executed by reactive engines (e.g., PlayGo [19]). The ability to execute these scenarios provides a platform to identify common elements in different scenarios or disagreements among them. The engines enable an incremental model to be formed in which scenarios can be added to an existing set (to learn more about LSCs and PlayGo visit http://www.weizmann.ac.il/mediawiki/playgo/index.php/Main_Page).

Putting medical records into LSC format involves translating the text into an object model consisting of a set of entities and events. The entities describe the participating components (i.e., doctor, patient and record) and the events describe the interactions among the elements. The object model describes a set of scenarios for the diagnosis and treatment of the patient, and the LSC formalism provides an interface that visualizes the scenarios. The modularity of the LSCs enables various aspects of disease development to be categorized. For example, it is possible to distinguish between the medical and psychiatric records, and to specify them as different scenarios. This enables medical records to be translated into a modular temporal description of disease development and allows the object model to grow incrementally when more scenarios and events are synthesized.

### Specification entities

The object model distinguishes between three key entities that actively participate in medical record information: the *Doctor*, the *Patient* and the *RecordPatient* (). The *Doctor* tracks the *patient* background, diagnoses the illness and documents CFS development and treatment in the *RecordPatient*. The patient reports his medical background and provides feedback concerning the efficacy of treatment(s), aiding the doctor in understanding the medical state. The Doctor-patient interaction is continuously documented in the *RecordPatient*. Each entity concerns an object and is represented in the GUI in the PlayGo tool.

**Figure 1:**
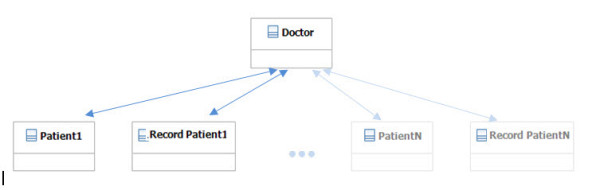
**Illustration of the interactions among the participating entities in the model.** A single doctor interacts with multiple patients and their medical records. Note that the patient may not access their record directly and must contact the doctor to modify/query personal data. However, the doctor may, in principle, query other patients or records to evaluate cases.

During the current phase of the object model development, a single *Doctor* diagnosed multiple patients. However, in principle, the object model enables multiple doctors to provide specifications for multiple patients, each with different medical conditions. Notably, the computational framework permits incremental processing of medical information during the formalization process, ensuring the formalized record is up to date after each doctor–patient session (see illustration in Figure 1).

### Specifying events between entities

LSCs are encoded using the Play-In technique, as implemented in the PlayGo tool. Play-In allows the user to create an LSC by clicking on the relevant elements in a graphical interface. User clicks describe desired events between entities in the system. The user enters the events by causing them to happen on the GUI. For example, if a user clicks the doctor entity and then the record entity, this defines an event between the two objects. The user can then enter the specific event from the existing list in the system, or alternatively can create an additional event to be added to the system. Each operation is automatically added to the LSC, which is generated on the fly, and in the continuously accumulating underlying model. Further details concerning play-in can be located in Additional file 1 and Additional file 2 at: http://www.weizmann.ac.il/mediawiki/playgo/index.php/Language_%26_Concepts#Play-In.

### Demonstration case: chronic fatigue syndrome (CFS)

CFS is a highly debilitating disorder with an unknown underlying cause. It is diagnosed by excluding medical and psychiatric diseases that can explain the symptoms reported by the patient. These symptoms include severe fatigue for six months or longer that is not relieved by rest, post-exertion malaise, impaired memory or concentration, un-refreshing sleep, muscle pain, multi-joint pain, tender lymph nodes, sore throat and headache [2]. As there are no objective diagnostic tests, physicians obtain extended medical information from patients in the form of interviews, medical records and health questionnaires. Treatment is aimed at relieving symptoms and often requires patients to visit a physician several times per year, resulting in the accumulation of extensive medical and management information for each CFS patient. Therefore, CFS is an interesting and challenging demonstration of how LSCs can be used to formalize this medical information.

### A conceptual example: LSC specification of a synthetic CFS report

**Figure 2:**
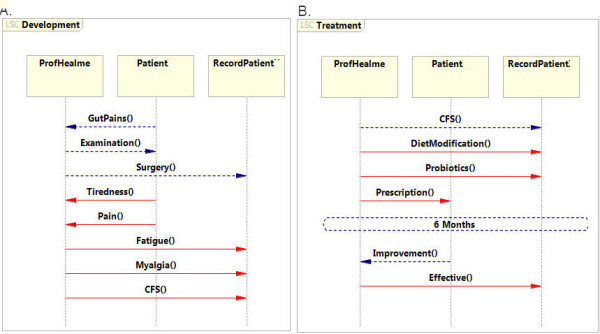
**A conceptual example of LSC for medical record: A**. LSC describing the development of the disease. **B**. LSC describing the treatment process.

To demonstrate the principle underlying the specification of medical information as LSCs, consider the following synthetic example and the accompanying LSCs specification depicted in Figure 2. While this synthetic case is a highly simplified version, it provides an example of how these types of records can be specified using LSCs.

In the synthetic case, CFS was diagnosed after gut surgery led to tiredness and physical weakness, causing pain and fatigue. Once CFS was diagnosed, the patient was treated with probiotics. After six months the patient reported improvements in the symptoms. To process the synthetic case into LSC specifications, key elements were extracted from the report. Each entry defines one event in the scenario; for example, the gut surgery is an entry in the scenario that describes an event in the development of the disease.

This synthetic record can be translated into two LSCs, one concerning the development of CFS (Figure 2A) and the other for treatment of the condition (Figure 2B). The *Development* LSC describes the event that led to the CFS diagnosis. The first set of events describes the development of CFS, with each element in the record having a designated event between participating entities (designated by italicized text). The patient visits the doctor and complains of *gut pains*, and after an *examination* undergoes *surgery*. Shortly after, the patient reports *tiredness* and *pain*, and the doctor documents *fatigue* and *myalgia*. Finally, based on the patient’s background, the doctor diagnoses *CFS*. With a diagnosis in place, the model switches to an alternate LSC called LSC *Treatment*. In this LSC the doctor recommends *diet modification* and *probiotics* for a period of *six months*. After this time period the patient reports *improvement* in his condition and the doctor records this.

### Specification of a CFS medical interview

To apply the methodology to CFS, an object model was formulated for the interview narrative obtained during a patient’s first visit. For confidentiality reasons the individual is referred to as patientX. The patientX interview narrative was specified as a set of LSCs to describe personal and medical background, and treatment. Further LSCs were added to ensure the specification was modular and readable, and to enforce the time dimension to describe the flow of events better. To share the object model with the scientific community and to allow more rapid development of a formalized database of medical information, the source code (Additional file 3) and instructions on how to install and use the model and the PlayGo tool (Additional file 1 and Additional file 2) are provided. Additional information can be located at: http://www.wisdom.weizmann.ac.il/~yaki/CFS.

The interview narrative was translated into distinct categories. The *Personalbackground* category consisted of personal patient information including age and place of birth. Subsequently, the details were formalized into *psychiatry* and *medical background* categories. Modularity was used to improve organization of the details, which were subcategorized into *diseases, injuries, surgeries and abnormal responses*. More categories and subcategories can be added if required to reflect additional information. In the case of CFS development, a newly defined category was added to emphasize details concerning the *fatigue* background that is central to the condition. The *fatigue background* category specifies relevant information concerning the influence of the fatigue related state and its causes. Furthermore, categories relating to *the past current and suggested treatments* were defined to describe the treatment the patient received and its effect. The set of categories defined in this object model is specific to this condition and this patient and can be altered to suit other illnesses; the formalism is flexible and allows additional categories to be added if required by the medical record.

### Doctor-patient inquiry and patient’s personal details

**Figure 3:**
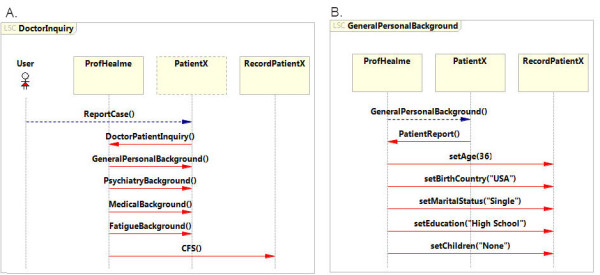
**Patient-Doctor inquiry and personal background. A**. LSC describing the general flow of events in the doctor’s report that contributed to a CFS diagnosis for patientX. **B**. The formalized personal background of PatientX, as extracted from the admission medical record.

The initial stage of the object model is the patient-doctor inquiry, which represents a doctor reporting to the database (Figure 3A). The *DoctorInquiry* LSC, and more specifically the *reportCase* and the *DoctorVisit* events, initiate the specification. The remaining events route entry through the various categories and subcategories of the interview narrative.

Four sequential events that are related to background categories describe aspects of the general background of the patient (*generalPersonalBackground* event), the general medical record (*medicalBackground* event), psychiatric background (*psychiatryBackground* event) and the specific fatigue background (*fatigueBackground* event). On the basis of these data, the doctor diagnosed patientX as suffering from CFS. The *GeneralPersonalBackground* LSC (Figure 3B) is initiated at the beginning of the doctor-patient inquiry (Figure 3A). This LSC details the personal background of the patient as described in the interview narrative. PatientX is 36 years old, was born in the USA and has a high school education. Therefore, the *generalPersonalBackground* LSC (Figure 3B), subsequent to the PatientReport event, defines events that indicate these details. This is described in five events: (1) setAge (“36”), (2) setBirthCountry (“USA”), (3) setMartialStatus (“Single”), setEducation (“HighSchool”) and setChildren (“None”).

### Psychiatric, medical and fatigue background

The object model presents the psychiatric background of the patient (Figure 2A). Each condition is described as an event with two parameters describing (1) the physiological condition and (2) the year it appeared. In this case, patientX had a nervous breakdown in 1987, was diagnosed with depression in 1996 and had a further nervous breakdown in 2003. Therefore, in the *PsychiatryBackground* LSC three events are specified that indicate the psychiatric condition of patientX. This is described in three distinct events: (1) ReportPsychiatryCondition (“NervousBreakdown”, 1987), (2) ReportPsychiatryCondition (“Depression”, 1996) and (3) ReportPsychiatryCondition (“NervousBreakdown”, 2003).

**Figure 4:**
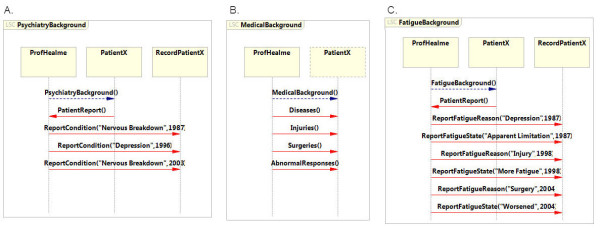
**Psychiatry, Medical and Fatigue Background specification for PatientX’s admission record. A**. LSC that specifies the psychiatric background of PatientX. **B**. LSC that specifies the subcategories of the medical background of PatientX. The specific information of each subcategory is given in a separate LSC and detailed in Figure 5. **C.** LSC that specifies the fatigue background of PatientX.

The specification follows with a description of the medical background of patientX (Figure 4B). This LSC extracts the relevant medical conditions that are not directly related to the fatigue record from the patient interview. The medical background was defined using subcategories: diseases, injuries, surgeries and abnormal responses record. For modularity of the LSC specifications, a separate LSC was dedicated to each subcategory. This layout provided a more modular specification that was easily created, read and debugged and traced.

First, the reported diseases and their duration were specified as a subcategory of the medical background, as a separate LSC (Figure 4A). Each disease is described as an event with three parameters: (1) the type of disease, (2) the age of the patient in which the disease was diagnosed, and (3) the duration of the disease (weeks). PatientX reported two diseases: rinderpest at the age of five years that lasted three weeks and an allergy that first appeared at the age of six and is ongoing. In the specification, these cases are translated to two events in the *Diseases* LSC. The first is *Disease (“Rinderpest”, 5, 3)* and the other is *Disease (“Allergy”, 6, -1)*. The −1 value in the second parameter indicates that the patient has never recovered from this affliction.

**Figure 5:**
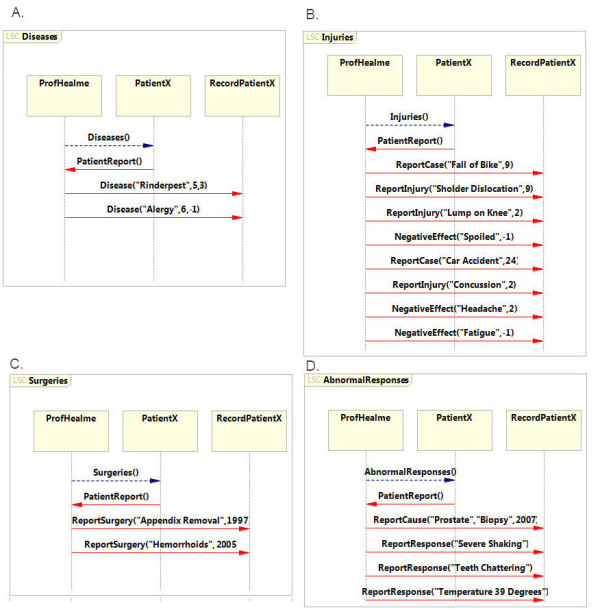
**Subcategories of the medical background. A**. Diseases of PatientX as specified in the LSC model. The scenario lists the patient’s diseases, the age of disease onset and disease duration. **B**. PatientX injuries as specified in the LSC model. The scenario includes information concerning past injuries and the effect they had on the patient. A described injury includes the year in which it happened, and a described effect includes the duration in weeks. **C**. PatientX surgeries as specified in the LSC model. The scenario lists the patient’s surgeries and the year for each surgery. **D**. The scenario includes information concerning causes of an abnormal effect, as reported by the patient and documented in the medical report.

Next, the lifespan injuries of patientX were described as an LSC using the injuries subcategory of the medical background (Figure 5B). As previously, the details from the medical record report were translated. For each injury the event of the injury and the resulting effect on the patient were described. In this specification each injury is defined using three events that describe (1) the case and the age of the event, (2) the effect of the injury and its duration and (3) the change in the patient’s condition and its duration.

In PatientX’s record two injuries are reported. First there was a bike accident at the age of nine that resulted in shoulder dislocation and a lump on his right knee that lasted for two weeks. This statement is translated to four events: (i) *ReportCase (“Fall off bike”, 9)*, (ii) *Injury (“Shoulder Dislocation”, 0)* (iii) *Injury (“Lump on Right Knee”, 2)* and *NegativeEffect (“Needy”, -1)*. Second, at the age of 24 patientX had a car accident and suffered minor concussion. The patient suffered from headaches for two weeks and has developed fatigue since. We defined the following events in the injuries LSC to describe the case: (i) *ReportCase (“Car accident”, 9)*, (ii) *Injury (“Concussion”, 0)* (iii) *NegativeEffect (“headache”, 2)* and (iv) *NegativeEffect (“fatigue”, -1).*

A similar approach was used to specify the surgeries patientX had undergone in a designated *Surgeries LSC* subcategory (Figure 5C). Each surgery and the year it was carried out are listed as an event. PatientX had two surgeries, appendix removal in 1997 and surgery for hemorrhoids in 2005. Therefore, in the specification after the report surgeries event, the LSC consists of two events: *ReportSurgery (“appendix Removal”, 1997)* and *ReportSurgery (“Hemorrhoids”, 2005)*. Lastly, the medical record LSC indicates the *AbnormalResponse* subcategory i.e., past medical trials that resulted in an abnormal response (Figure 5D). In the case of the LSC pertaining to patientX two types of events are specified: (1) the event that caused the response followed by (2) the list of abnormal responses. In the medical record of patientX an abnormal response was recorded after a biopsy in 2007. The trial resulted in severe shaking, teeth chattering and a temperature of 39°C. This is indicated in the *AbnormalResponse* LSC using four events: one indicates the case, *ReportCause (“Biopsy”, 2007)*, and three events describe the responses, (i) *ReportResponse (“Severe Shaking”)*, (ii) *ReportResponse (“Teeth Chattering”)* and (iii) *ReportResponse (“Temperature 39°C”)*.

From the doctor-patient inquiry, relevant details concerning the fatigue background are documented in a separate LSC using two distinct events: (1) an event describing the reason for the change in the fatigue condition and (2) the effect on the fatigue state. Each event has an additional parameter that indicates the year the case occurred. PatientX had three cases that affected the fatigue state; the fatigue initiated after depression in 1997 that led to activity limitation, an injury in 1998 that increased fatigue and surgery in 2004 that worsened the condition. This is indicated in the *FatigueBackground* LSC in three clusters of events, each consisting of the cause and effect (Figure 4C): (1) *ReportFatigueReasons (“Depression”, 1987)* and *ReportFatiqueState (“Apparent Limitation”, 1987)*, (2) *ReportFatigueReasons (“Injury”, 1998)* and ReportFatiqueState *(“More Fatigue”, 1998)*, and (3) *ReportFatigueReasons (“Surgery”, 2004)* and ReportFatiqueState *(“Worsened”, 2004).*

### Diagnosis and treatments

The *DoctorInquiry* LSC indicates at the final event the diagnosis with the designated *CFS* event (Figure 3). This event directs the specification to a set of LSCs that describe the treatment history of patientX. The specification covers the treatment using four distinct categories: (1) treatments that were proven ineffective, (2) treatments that had a negative effect, (3) present treatment and (4) suggested treatment.

**Figure 6:**
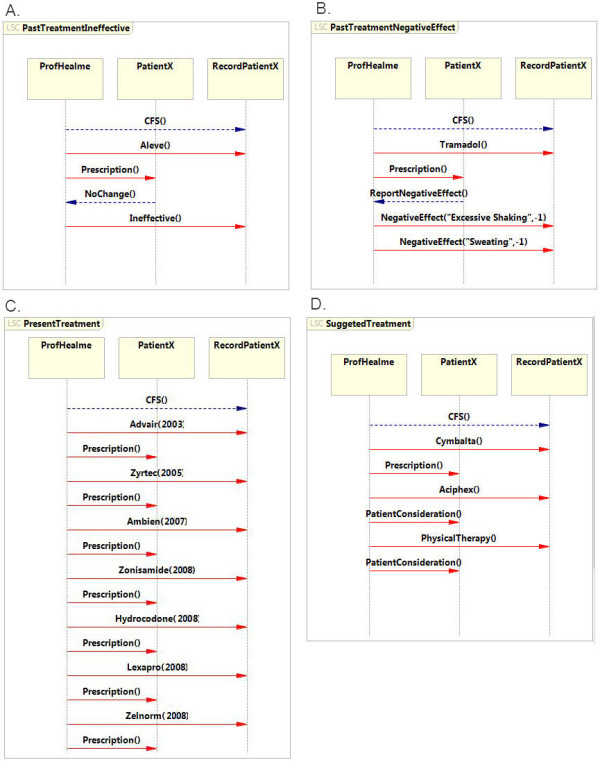
**Specifications of PatientX’s treatment extracted from the admission medical record. A**. LSC describing PatientX’s ineffective treatments. **B**. LSC describing PatientX’s past treatments that caused a negative effect. **C**. LSC describing PatientX’s current medications. **D**. LSC describing PatientX’s suggested treatment.

In the case of patientX, the *PastTreatmentIneffective* LSC (Figure 6A) consists of a single treatment prescribed to the patient that had no effect and was reported ineffective. This is indicated in the LSC by four events. First, the doctor suggests Aleve as a treatment and documents this in the records (*Aleve ()* event). This is prescribed to patientX (*Prescription* () event). PatientX reports that Aleve has no effect (*NoChange* () event) and the doctor records this in the record (*Ineffective* () event). Similarly, medicines that led to a negative effect are indicated as events in the *PastTreatmentNegativeEffect* LSC. However, in the case of negative effects, additional events describe the effect of the medicine on the patient’s health (Figure 6). The medical record of patientX reports a negative response of excessive shaking and sweating that lasted as long as Tramadol was taken. In the *PastTreatmentNegativeEffect* LSC the event *Tramadol ()* and the subsequent *Prescription ()* are followed by a *reportNegativeEffect () event* from the patient to the doctor. The doctor records the negative effects in two events *NegativeEffect (“Excessive Shaking”, -1)* and *NegativeEffect (“Sweating”).*

The last two LSCs in this specification indicate the current treatment and the suggested treatment for patientX. The *CurrentTreatment* LSC consists of events for each medication and the year it was prescribed (Figure 6C). For example, Advair has been administered regularly to patientX since 2003. Therefore, the specification consists of the event *Advair (2003)* and a subsequent *perspiration ()* event. Similar events describe other medications that patientX uses. The suggested treatment is described in the *suggestedTreatment* LSC (Figure 6D), where the treatment can be followed by a *prescription ()* event or by *patientConsideration () event.* The former indicates that the doctor has prescribed the medication to the patient but did not instruct the patient to take it. The latter indicates that the doctor mentioned the possibility to the patient, but suggested the patient report his current condition before the medication was prescribed.

### A formal designated event vocabulary for CFS development

This object model consists of a centralized perspective from which the user can view and manage events and entities in the model. In the context of CFS and disease development, this centralized perspective provides an event vocabulary that is dynamically defined and grows. When a new event is entered (e.g., a patient reports a new symptom), it is translated to an entry that immediately appears in the vocabulary. For example, if a strong pain in the left arm is reported a ‘painInLeftArm’ event would be added to the system model and used in the LSC. Next time an individual (not necessarily the same patient) reports pain in their left arm the event will be in the vocabulary and can be used. The fact that a certain event exists or does not exist in the vocabulary provides an indication concerning the type of events experienced by CFS patients. Future development of an automated parser may be indicated, which would parse electronic medical records (EMRs) into LSC specification. This activity would be complementary to using text mining and natural language techniques in medical research [7,9].

**Figure 7:**
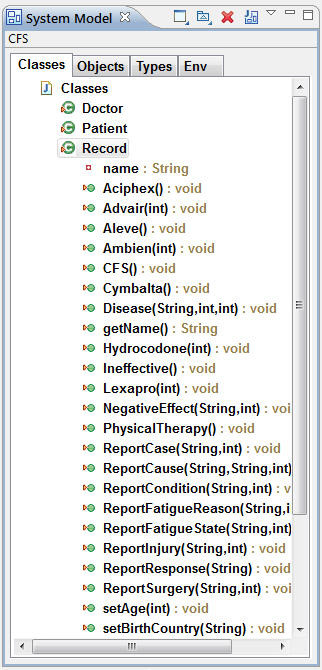
**A vocabulary for CFS development as specified by the object model.** The vocabulary consists of the terminology used throughout the object model and can be used for future specification of medical records.

The event vocabulary consists of two types of events: Generic and Specific. *A generic event* describes a general event during CFS development, and a *specific event* designates a concrete element. A general event has parameters that add further details to the case, for example: the fatigue condition is defined by the change in the fatigue state and the year in which the patient experienced the event. Therefore, the fatigue condition has two parameters: (1) state and (2) year. A *specific event* is defined by specific data including the medicine that was prescribed to a patient. In this example, the vocabulary aims to include various medicines administered to treat CFS as specific events. In contrast, when considering medical background, events for the fatigue state are generic in the vocabulary. Therefore, throughout the specification the various fatigue states are documented using *reportFatigueState (“apparent limitation”, 1987) event*, whereas the *Advair ()* medicine event is defined specifically in the vocabulary. A partial list of the vocabulary is presented in Figure 7.

This vocabulary can be analyzed programmatically. This is a valuable capability that allows utilities to be added as required; for example, exporting a list of medicines related to CFS, or for systematic searching. A new event in the vocabulary can define a future requirement and can be broadcasted as a message to a central CFS database. The PlayGo tool supports a technique termed Play-in [20], where the user can amend the vocabulary in an intuitive way by clicking on the relevant entity. Therefore, as a user of the database, the doctor can extend the vocabulary with a click of a mouse.

## Conclusions

Medical information, in the form of an interview narrative, can be synthesized into a rigorous formalism. The output is an object model that provides a description of the medical narrative as a collection of modular scenarios. Potentially, this provides a powerful method of analyzing interrelations among disease development, diagnosis and treatment. We are currently in the process of specifying a database of CFS medical interview narratives and records to construct a formal scenario database. The database would be continuously updated with new patients and their associated medical records.

During the first stage, LSC is encoded manually from the record to the scenario. The modeler is required to identify the key entries for each case and to translate them to events. However, as the modeling process advances and the event vocabulary is set, specification will be directed by the set of the dictionary entities. The PlayGo platform stores scenarios, prevents typographical errors and allows identical events to be handled in different scenarios. Therefore, records are never excluded from the database. Future extensions could automatically translate the medical records into LSC specifications. In the long term, a specific scenario view plugin could be developed to display the scenario of each record.

The scenario database could serve as a platform for systematic analysis of disease development in numerous patients. Possible analysis and data mining directions include mining the LSCs scenarios to discover motifs and development patterns, analyzing correlations between scenarios in different patients at the pre-diagnosis stage and disease development. For example, LSC databases could aid in the evaluation of the effectiveness of treatments i.e. an event (or set of events) in the diagnosis scenario that is correlated with a successful or unsuccessful treatment. Furthermore, the database could serve to investigate interrelationships and common patterns between different patients with the same diagnosis, and could highlight if several scenarios repeat a similar pattern. Mining and analysis of the database could be carried out using tools that are currently widely used in bioinformatics. However, owing to the lack of a proper platform, most of these tools have rarely been applied to medical data or to systematic study development and diagnosis of medical records.

The methodology presented here could assist the future development of ‘virtual patients’. The nature of the PlayGo tool enables the LSC specification to be executed, allowing tests concerning whether the conditions reported by a new patient have been previously reported. Therefore, in principle, the formalized database of the medical record can be embedded behind an interface that allows a patient to input current conditions. The software would translate the user input into LSC specification and run it against the database. Once a match was found, the system returns the treatment that was given and its effectiveness. This platform could have a twofold impact, as it evaluates the probability of a patient having a particular diagnosis and assists the clinician/user in learning about similar cases and the way in which they were treated.

## Abbreviations

LSC: Live sequence charts; CFS: Chronic fatigue syndrome.

## Competing interests

The authors declare they have no competing interests.

## Authors’ contributions

YS conceived and coordinated the study and wrote the paper. YS analyzed the medical records and designed the LSCs. YS and SS implemented the model. EA, SDV and LB provided the medical information and advised on medical relevance. JB advised on paper format and content. All authors read and approved the final manuscript.

## Supplementary Material

Additional file 1 **Instructions on how to install and use the model and the PlayGo tool.** (DOCX 532 kb) Click here for file

Additional file 2 **Instructions on how to specify scenarios (the play-in process).** (DOCX 1043 kb) Click here for file

Additional file 3 **The source code of the LSC model for the CFS medical record.** (UML 244 kb) Click here for file
