# Supplementary material for: Live sequence charts to model medical information
Source: Theor Biol Med Model. 2012 Jun 15;9:22. doi: 10.1186/1742-4682-9-22 (PMC3536704; doi:10.1186/1742-4682-9-22)

### Install PlayGo

Download PlayGo from: <http://www.weizmann.ac.il/mediawiki/playgo/index.php/PlayGo_IDE>.

Extract the downloaded zip file to a newly created directory. Throughout this document, this directory is referred to as <PlayGo>.

Once extracted, the <PlayGo> directory should have the following content:


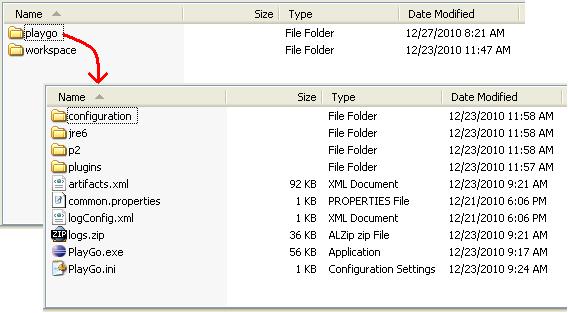


### Startup PlayGo

To start PlayGo, go to the <PlayGo>/playgo/ directory and double-click the PlayGo.exe file. As it is in Eclipse, PlayGo will ask you for the workspace to start with. Browse to the new workspace location and click OK.

### Configure PlayGo

The following settings should be configured before you start working:

1. From the Window menu, choose Preferences->Google->Web Toolkit. Configure it to point to the <PlayGo>\plugins\com.google.gwt.eclipse.sdkbundle.2.0.0_2.0.0.v200912062003\gwt-2.0.0 directory:


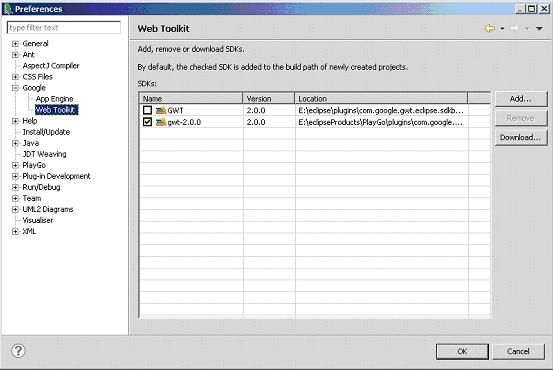


2. From the Window menu, choose Preferences->Google->App Engine. Configure it to point to the <PlayGo>\plugins\com.google.appengine.eclipse.sdkbundle.1.3.0_1.3.0.v200912141120\appengine-java-sdk-1.3.0 directory:


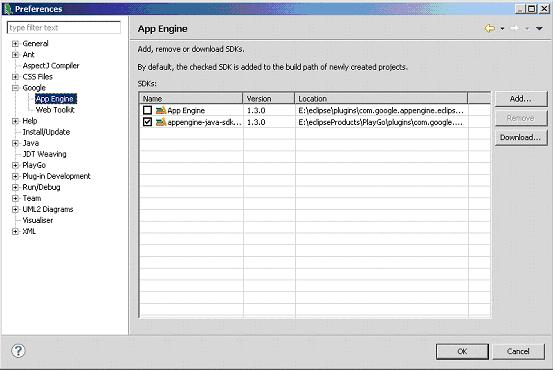


### Create CFS project

1. Download the CFS model from <http://wisdom.weizmann.ac.il/~yaki/CFS>.
2. From the file menu choose: New --> LSC Project. Note: if you are not using the PlayGo perspective you may not see this option. In such cases choose New --> Other --> PlayGo category --> LSC Project.
3. Provide a project name (e.g. MedicalRecord).
4. Select the ‘Choose model’ option and browse to select the CFS model that was downloaded in Step 1.
5. Press the Finish button.


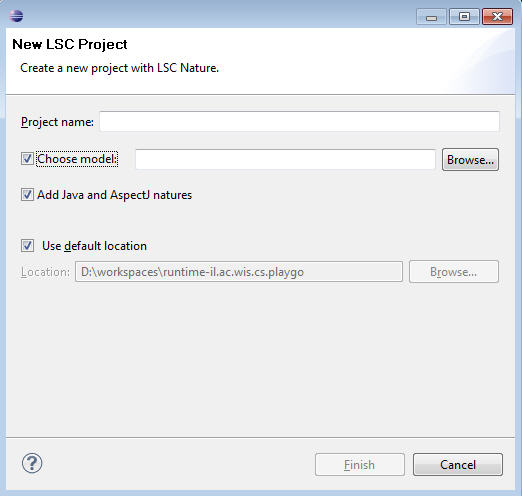


A new project was created (which you can browse from the left side panel) with the following structure:


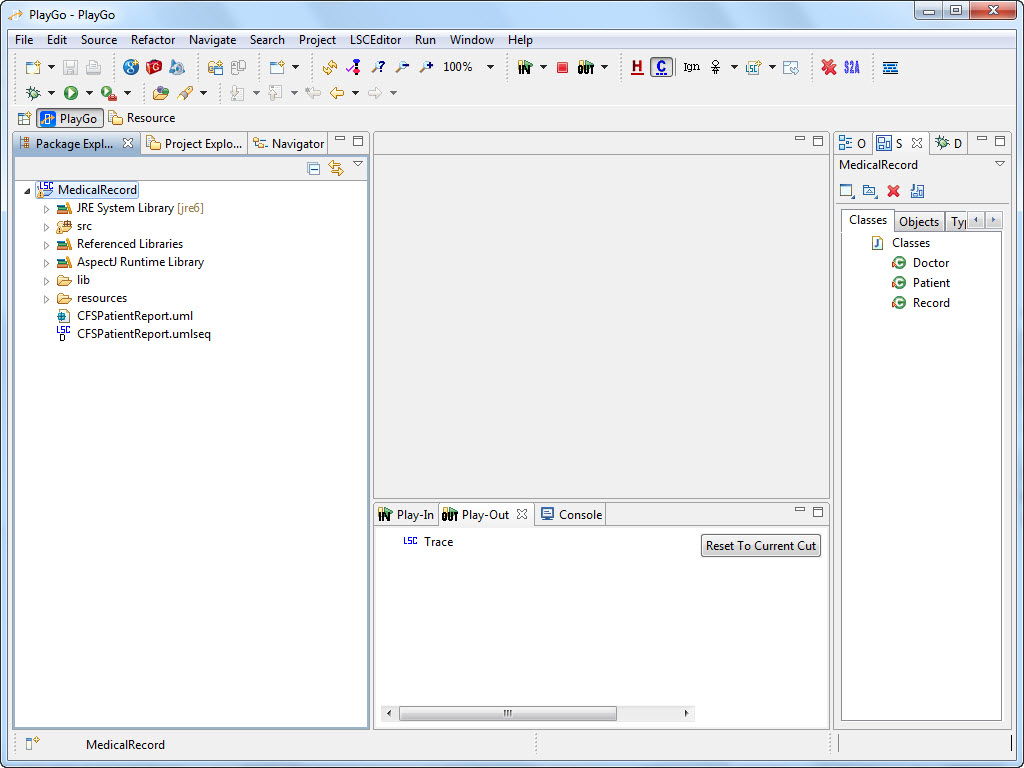


### View the CFS model

To view the CSF Model, double click the CFSPatientReport.umlseq file. An LSC editor will open showing the list of LSCs:


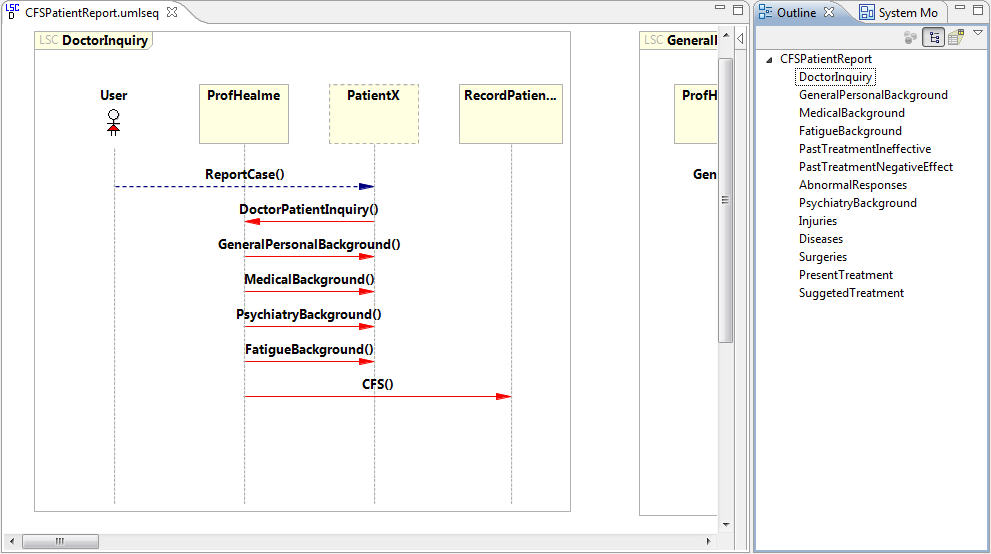


### Play-out the CFS model

- Select the project you’ve created (e.g. MedicalRecord project) and click the play-out icon on the toolbar:


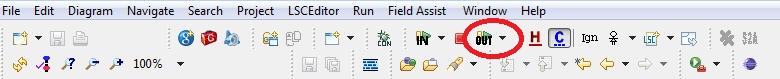


- Open the System Model view (if the view is not opened, go to: Windows -> Show View -> Other… -> PlayGo -> System Model)
- Select the ‘Objects’ tab and expand the PatientX object
- Start the execution by right-clicking the ReportCase() method and selecting ‘Call Message’
- You can follow the cut change in the Play-Out view at the bottom of the workbench:


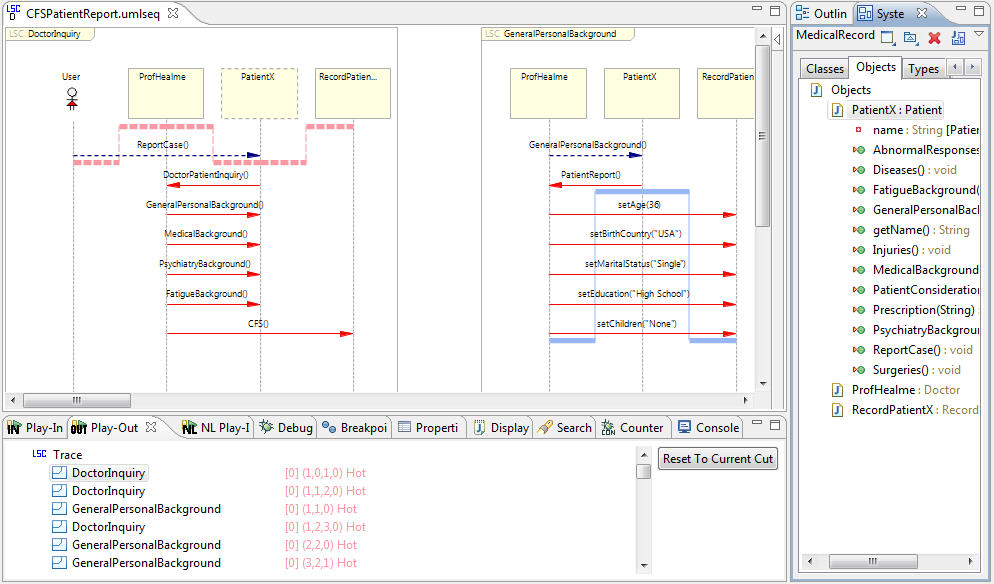

Supplement: Additional file 1 — Instructions on how to install and use the model and the PlayGo tool. (DOCX 532 kb) [file 1742-4682-9-22-S1.docx]
