# Supplementary material for: Live sequence charts to model medical information
Source: Theor Biol Med Model. 2012 Jun 15;9:22. doi: 10.1186/1742-4682-9-22 (PMC3536704; doi:10.1186/1742-4682-9-22)

How to specify LSCs
(the Play-in process in PlayGo)

# Overview

This document describes how to specify LSCs for medical records. PlayGo, a reactive engine that supports LSC specification and execution, allows this using a technique termed ‘Play-in’. The Play-in process is explained and implementation of CFS medical records in PlayGo is illustrated. More general information concerning PlayGo and instructions relating to the installation process can be obtained at: [www.playgo.co](http://www.playgo.co). The source code for the complete CFS model and instructions on its use in PlayGo can be obtained at: <http://www.wisdom.weizmann.ac.il/~yaki/CFS/>.

# *Play-in* medical records in PlayGo

To specify the medical record in LSC using PlayGo, the user defines a system model and the relevant operations. Herein, the creation of a sample LSC scenario using PlayGo, as appears in the main manuscript, is demonstrated. The LSC scenario we focus on in our explanation is the following:


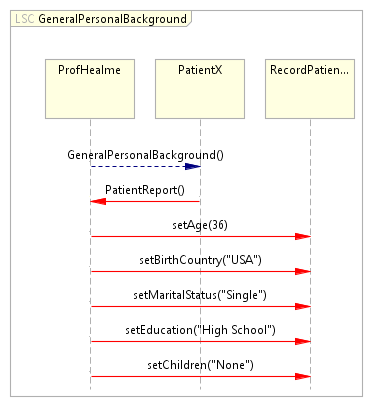


We start by demonstrating how to create a system model:

1. Create a new LSC project, or upload the CFS project supplemented to the paper.
2. In the ‘System Model’ view select the ‘Classes’ tab:


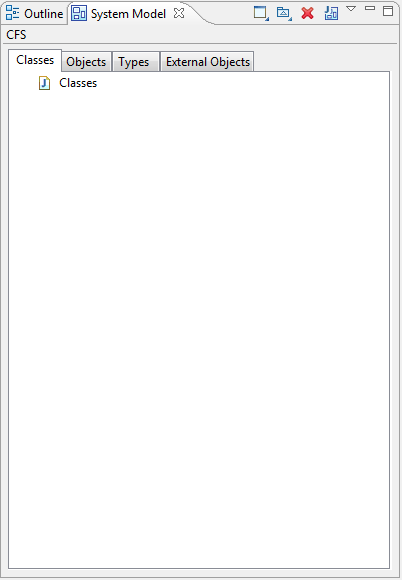


1. To add a new class, select the classes root entry, right-click it, and select ‘Add Class’:


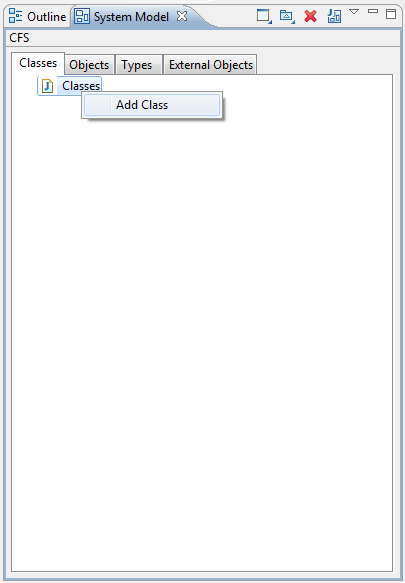


1. Type the Name of the class (the other fields are irrelevant for the scope of this project), for example: the following snapshot defines the class Doctor, which represents the medical authority that treats patients:


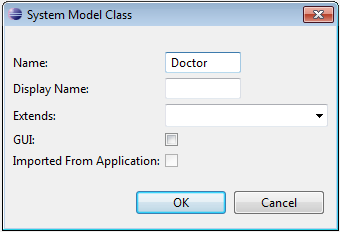


1. Each possible action specified for the object is defined as a specific method. In our example, a method is defined for each action of a Doctor class (e.g., the method Report Negative Effect specifies the action of a doctor reporting a negative effect). To add a method, right-click on the class name and chose ‘Add Method’ from the menu list:


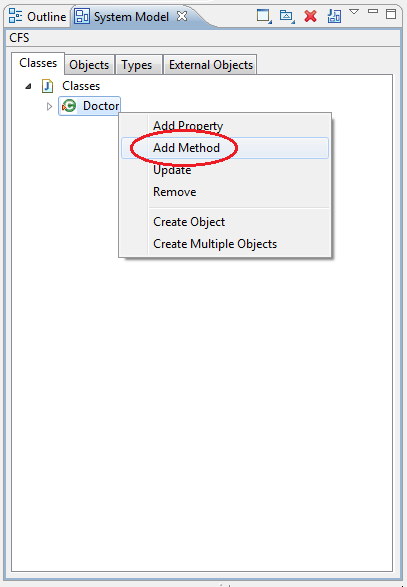


1. Enter the details of the method/action (in case the method carries argument, fill the list of argument by types separated by a comma; the remainder of the entries are irrelevant in the scope of this model):


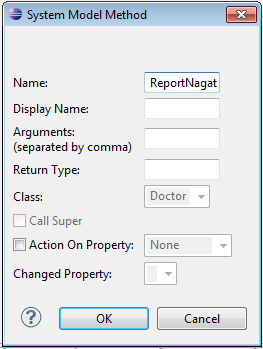


1. Repeat steps 5 and 6 for each activity of the class (in this case the actions that are specified for the Doctor).
2. Once the class and its methods are defined, we shell create instances of the class to define the specific elements that participate in the model, for example: ProfHealme is an instantiation of object of class Doctor. To create an object, right-click on the class name and chose ‘Create Object’ from the menu list:


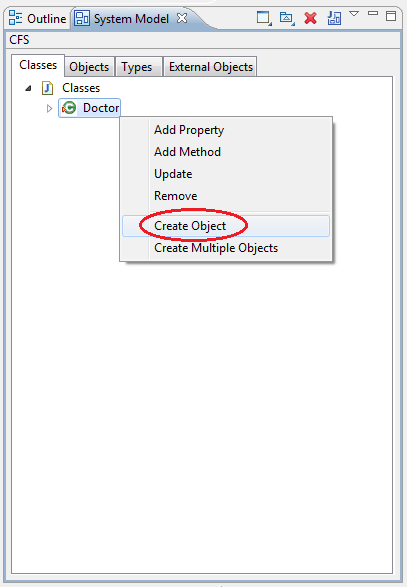


1. Repeat steps 1-8 for each new class in the system.

In the CFS example, three classes were defined; one is the Doctor class, the second is the Patient class and the third is the Record class. Each class comprises multiple methods, as demonstrated in the figure below. For each class one instance was defined (i.e., Prof Healme is an instance of the Doctor class; PatientX is an instance of the Patient class and Record PatientX is an instance of the Record Class).


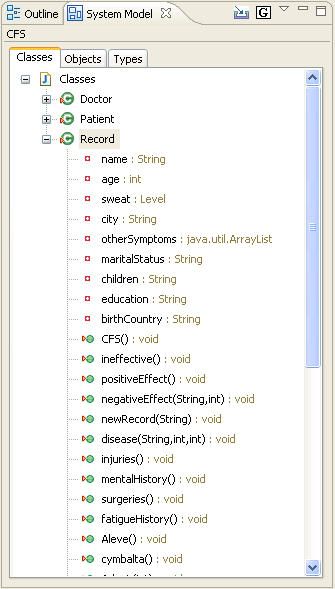


Once the system model is created, defining the classes and methods in the system, *play-in* is used to translate the medical record to LSC (i.e., specifying system behavior). It is a requirement to have a defined system model for the *play-in* process. However, the user can halt the *play-in* process at any time to update the system model with more information.

As part of the play-in process events between participating objects are defined. Events are chosen from the method list defined in the object’s class. We distinguish between two main event-types. The first event-type designates events that trigger the LSC. These ‘cold monitor’ events, which may or may not take place, are visualized by a blue dashed arrow. The second event–type, visualized by a red solid arrow, are events that occur once the LSC scenario is triggered. These are ‘hot execute’ events that must take place in the LSC scenario. For example: the ‘General Personal Background’ in bullet 1 of the LSC scenario, is defined as a ‘cold monitor’ event. Once this event is triggered the remaining ‘hot execute’ events (e.g., patient Report) must subsequently be triggered.

The steps required to define the ‘General Personal Background’ event are detailed below, illustrating how to create a ‘cold monitor’ event.

1. Click the ‘cold/monitor’ button on the toolbar:


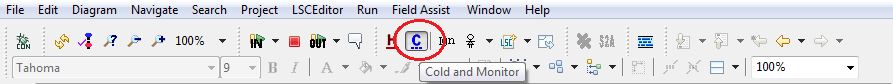


1. In the system model view, select the ‘Objects’ tab:


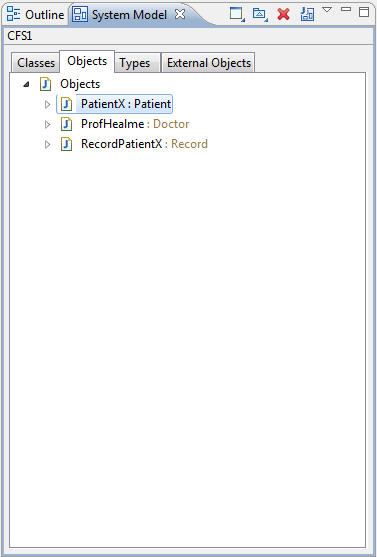


1. Select the object ‘ProfHealme’; right-click it and choose ‘Call Other Object’:


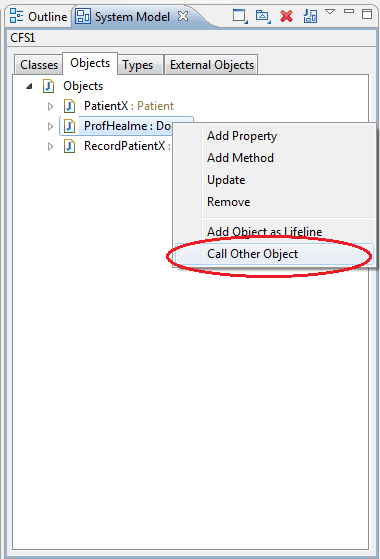


1. Select the object PatientX and expand it by clicking the small arrow or plus sign at the left-hand side. This allows you to see all operations/events that are available for PatientX:


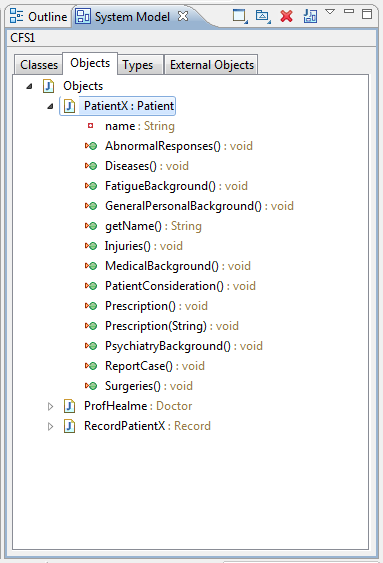


1. Select the method ‘GeneralPersonalBackground’, right-click it and select ‘Call Message’. The method may require additional arguments. Therefore, PlayGo prompts the user to open a specific window where values for the parameters can be set.


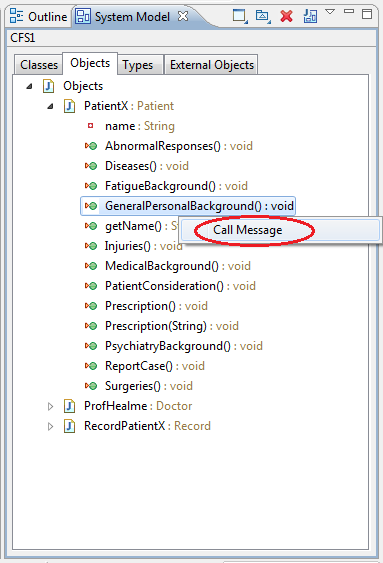


1. As a result, an event that connects the two objects appears in the LSC scenario. The event is visualized as a blue arrow labeled ‘GeneralPersonalBackground’, with the ProfHealme object as the source and the PatientX object as the target.


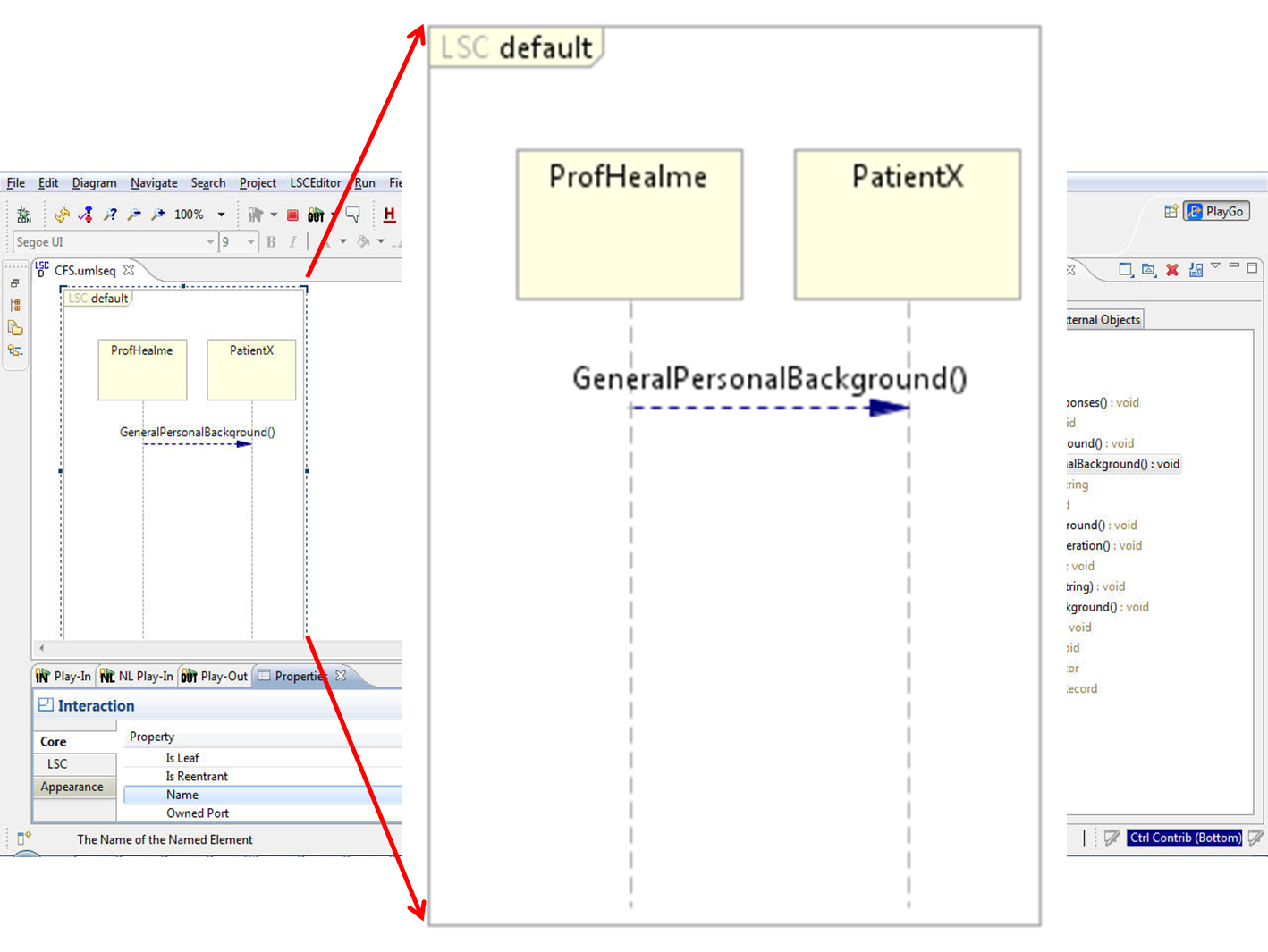


The default property values can be modified by setting the ‘Properties’ view at the bottom of the canvas.

Similar steps are required to play-in ‘hot execute’ events. Details concerning how to play-in the ‘PatientReport’ event, a representative example, are detailed below.

1. Click the ‘hot/execute’ button on the toolbar:


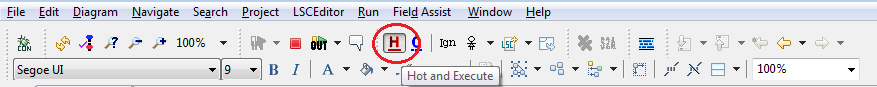


1. In the system model view, select the ‘Objects’ tab:


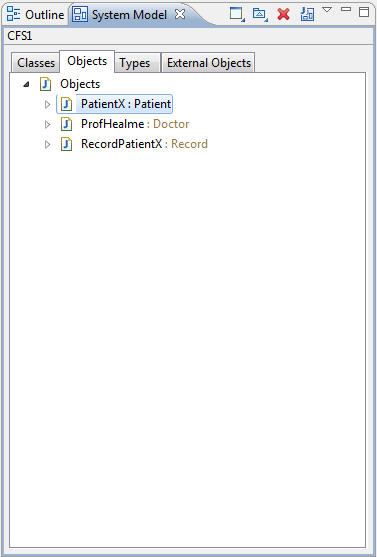


1. Select the object ‘ProfHealme’, right-click it and choose ‘Call Other Object’:


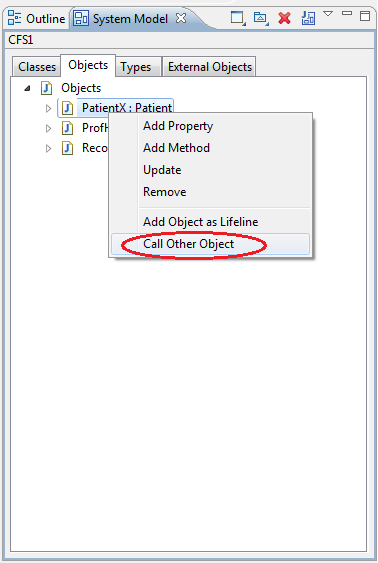


1. Select the object ProfHealme and expand it by clicking the small arrow or plus sign at the left-hand side. This allows the user to see all operations/events that are available for ProfHealme:


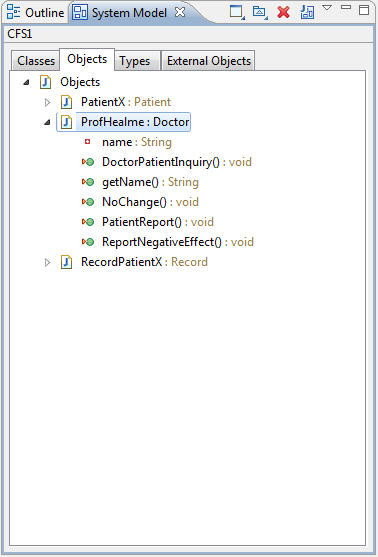


1. Select the method ‘PatientReport’, right-click it and select ‘Call Message’. The method may require additional arguments. Therefore, PlayGo prompts the user to open a specific window where values for the parameters can be set.


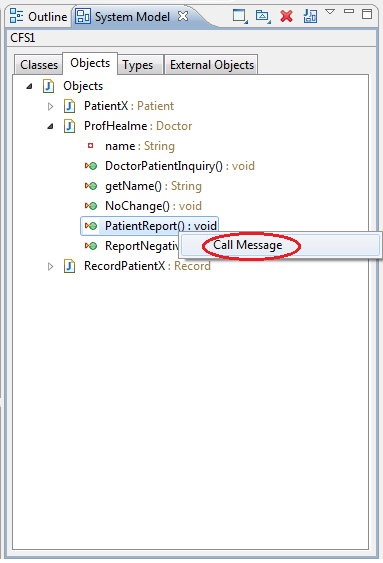


1. As a result, an event that connects the two objects appears in the LSC scenario. The event is visualized as a red arrow labeled ‘PatientReport’, with the PatientX object as the source and the ProfHealme object as the target:


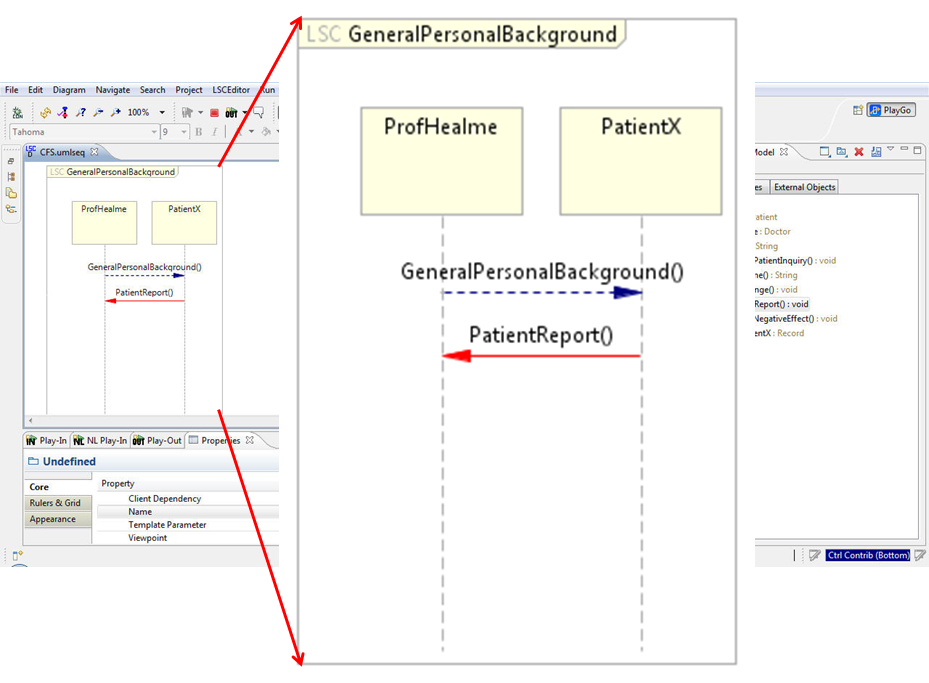


The rest of the events can be added to the LSC in a similar manner.


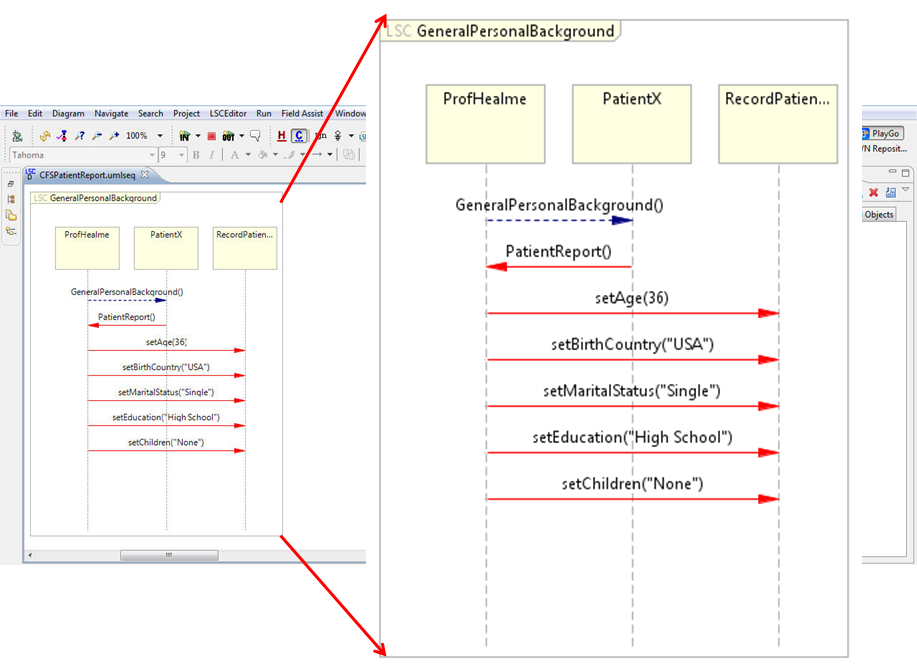

Supplement: Additional file 2 — Instructions on how to specify scenarios (the play-in process). (DOCX 1043 kb) [file 1742-4682-9-22-S2.docx]
